# Food insecurity among women of reproductive age in Nepal: prevalence and correlates

**DOI:** 10.1186/s12889-020-8298-4

**Published:** 2020-02-04

**Authors:** Shanta Pandey, Vincent Fusaro

**Affiliations:** Boston College School of Social Work, McGuinn Hall, Room 311, 140 Commonwealth Avenue, Chestnut Hill, MA 02467 USA

**Keywords:** Food insecurity, Women of reproductive age, Nepal

## Abstract

**Background:**

Food insecurity is widely prevalent in certain sections of society in low and middle-income countries. The United Nations has challenged all member countries to eliminate hunger for all people by 2030. This study examines the prevalence and correlates of household food insecurity among women, especially Dalit women of reproductive age in Nepal.

**Methods:**

Data came from *2016 Nepal Demographic Health Survey,* a cross-sectional, nationally representative survey that included 12,862 women between 15 and 49 years of age of which 12% were Dalit. Descriptive analysis was used to assess the prevalence of household food insecurity while logistic regression examined the relationship between women’s ethnicity and the risk of food insecurity after accounting for demographic, economic, cultural, and geo-ecological characteristics.

**Results:**

About 56% of all women and 76% of Dalit women had experienced food insecurity. Ethnicity is strongly related to food insecurity. Dalit women were most likely to be food insecure, even after accounting for factors such as education and wealth. They were 82, 85, 89 and 92% more vulnerable to food insecurity than Muslims, Brahmin/Chhetri, *Terai* Indigenous, and Hill Indigenous populations, respectively. Education was a protective factor—women with secondary education (6th to 10th grade) were 39% less likely to be food insecure compared to their counterparts without education. With a more than 10th grade education, women were 2.27 times more likely to be food secure compared to their counterparts without education. Marriage was also protective. Economically, household wealth is inversely correlated with food insecurity. Finally, residence in the Mid-Western, Far-Western and Central Development regions was correlated with food insecurity.

**Conclusion:**

To reduce food insecurity in Nepal, interventions should focus on improving women’s education and wealth, especially among Dalit and those residing in the Far- and Mid-Western regions.

## Background

Household food insecurity (HFI) refers to the lack of consistent household access to an adequate quantity of healthy foods [1–3]. It is also an indicator of economic deprivation, symptomatic of insufficient economic resources to meet basic needs. Food insecurity is widespread; in 2016, about 815 million people were chronically hungry and undernourished worldwide [4, 5]. It also has serious consequences for wellbeing. HFI is associated with adverse nutritional, physical and mental health outcomes for both children and adults [4, 6–9]. While all food insecurity is concerning, the implications for women of childbearing age are particularly acute due to the potential for intergenerational consequences.

The consequences of HFI are many. HFI affects women’s and children’s diet diversity which in turn shapes their nutritional outcomes [10]. Women and children in food-insecure households often consume fewer fruits, vegetables, nuts, and protein-rich foods but more refined grains, bread, and sweets; they are therefore at risk for obesity and diabetes [11–13]. Malnutrition before and during pregnancy is associated with a range of health problems for the mother and the developing fetus [14]. Food insecurity also increases the risk of anxiety and depression in mothers [15] and reduces the likelihood of breastfeeding [16]. In a study in Zimbabwe, maternal food insecurity was associated with an increased risk of *human immunodeficiency virus (*HIV) infection and a greater subsequent risk for failure to access services to prevent mother-to-child transmission [17, 18]. In Mexico, women of reproductive age from food insecure households were more likely to experience anemia and be overweight due to malnutrition [19]. In Quito, Ecuador women with children from food insecure homes reported poorer self-rated health and higher mental health problems, including stress and depression [20]. In northwest Ethiopia, HFI was associated with undernutrition in mothers with children under five years of age [21]. HFI also adversely affects child wellbeing. Children from food insecure homes are more likely to suffer from stunting, underweight, and wasting [7–9, 22–25]. In rural Indonesia, HFI was correlated with higher neonatal, infant and under-five child mortality [26].

Researchers have identified some of the determinants of HFI. Studies from around the world show that food insecurity is associated with having low levels of education, weak social networks, less social capital, low household income, and being unemployed [13, 27–29]. In Lebanon, household income and women’s education were inversely associated with HFI [11]. In Uruguay and Brazil, household income strongly correlated with food insecurity [30, 31]. Among people living with HIV in Nigeria, food insecurity was associated with educational attainment, occupation, and living conditions such as housing and property ownership status [32]. Social structures also play a major role in food insecurity. For example, female-headed households are at no greater risk for food insecurity than male-headed households when there is relative social equality between genders [33].

In sum, existing literature has identified several demographic and socioeconomic factors predicting HFI across many developing countries. In this paper, we focus specifically on predictors of HFI among women, especially Dalit women of child-bearing age in Nepal. While one would expect many of the risk factors found in other contexts to apply to Nepal, there are additional issues–caste-based systematic exclusion and geo-ecological factors—that make it worth particular examination. Social exclusion of an individual from participating in key socio-economic opportunities of the society in which he or she lives contributes to unequal access to resources [34]. In turn, some segments of society are unable to realize their full potential [34, 35]. In Nepal and India, the Dalits—formerly referred to as “untouchables”— belong to the lowest rungs in the Hindu caste system and have a long history of marginalization from educational or employment opportunities [36–39]. Although caste-based discrimination of the Dalits was legally abolished in 1963 and the Maoist Movement between 1996 and 2006 helped dissipate some of the caste-based discriminatory practices, socio-economic marginalization of the Dalits in Nepal continues [39]. Even accounting for other factors, the legacy of the caste social structure may increase the risk of food insecurity for this group. Hence, it is important to document the magnitude of food insecurity among Dalit women of reproductive age compared to other ethnic groups.

In Nepal, geographic and ecological factors also contribute to the isolation of people from some regions from realizing the benefits of development opportunities and exacerbate their HFI. Previous research has documented that HFI is more prevalent in the Western, Mid-western and Far-Western regions of Nepal [8, 9, 40]. A detailed study in Dailekh District, Mid-Western Development Region showed that 75% of households were food insecure, with 23% chronically food insecure and 52% seasonally food insecure 38. As expected, this study also found that HFI was substantially more common among Dalits and the poor—such as small landholders and day laborers—generally. HFI fluctuated seasonally and was more noticeable in nearly every household in Dailekh District from late June to early August and late February to early April. This seasonality coincides with crop harvests—wheat in late April, maize in late August, and rice in late November [38]. Another study using 2011–12 agricultural census data examined HFI in three eastern districts of Nepal: Taplejung, Panchthar, and Jhapa. It found that households experienced food insecurity for about 3.5 months in a year and, underscoring the current focus in women of child-bearing age, that female-headed households were significantly more food insecure than male-headed households [41].

These studies either rely on local data to understand the level of household food insecurity8,38,41 or use 2011 Nepal Demographic and Health Survey data to study the consequences of food insecurity on health behavior [40] or nutritional status of children and women [9]. The current study uses the 2016 nationally representative data and asks the following questions: How prevalent is food insecurity among women of reproductive age in Nepal? Is the risk of food insecurity higher for Dalit women compared to other ethnic groups after accounting for their demographic, economic, cultural and geo-ecological indicators?

## Methods

This cross-sectional study examined demographic, socio-economic, and geographic determinants of food insecurity in Nepal. We used data from the *2016 Nepal Demographic and Health Survey (NDHS)*, a nationally representative, comprehensive survey administered between June 2016 and January 2017 [42]. The survey used a two-stage (in rural areas) and a three-stage (in urban areas) stratified sample design. Data were collected from all women between 15 and 49 years of age in 11,040 households, resulting in a sample of 12,862 women of reproductive age. The entire questionnaire is published at the end of the 2016 NDHS report (available from, https://www.dhsprogram.com/pubs/pdf/fr336/fr336.pdf) [42].

### Measures

The dependent variable is a dichotomous indicator of food insecurity measured by the nine-item Household Food Insecurity Access Scale (HFIAS). A team of researchers at Tufts University originally developed this Scale in 2006 to assess household food insecurity in developing countries [43, 44]. In general, this scale assesses the household head’s experience of food insecurity within 30 days. When launching the scale in developing countries, a one-year recall accounts for the harvests of various crops. Multiple studies in developed and developing countries have validated the HFIAS [45–52]. In the current study, the HFIAS assesses the household head’s experience of food insecurity in the twelve months before the survey interview. Questions cover topics such as whether the respondent was worried the household would have sufficient food, eating undesired foods because of a lack of alternatives, and whether a member of the household ever went an entire day without food because of lack of food availability. The answers for each item included: never (coded as 0), rarely (coded as 1), sometimes (coded as 2), and often (coded as 3). A reliability test using the Nepal data showed that the nine-item food insecurity scale had a Standardized Chronbach Alpha coefficient of .90 indicating excellent internal consistency. Summed responses to the scale ranged from 0 to 27, with 44.26% answering never (0) to all nine questions.

Previous studies using the HFIAS scale have either used the summated scale score as a continuous variable or divided the scores into four categories—food secure households (those scoring 0 in the summated scale), mildly food insecure (those scoring 1 or 2 points), moderately food insecure (those scoring 3 to 10 points) and severely food insecure households (those scoring more than10 points) [46, 49]. In these studies, the food insecurity experience recall period was 30 days. The current study has a recall period of 12 months. The distribution of the scale score was nearly bimodal, with 44.26% scoring zero and indicating these households were food secure throughout the year, 12% being mildly food insecure, 36.33% moderately food insecure, and 7.41% severely food insecure households. Given this distribution and the recall period of 12 months, a binary variable comparing those who were clearly food secure throughout the year with those who were food insecure would be more useful in understanding HFI in Nepal. Hence, to create the dependent variable, women who answered “never” to all items were considered food secure throughout the year and were coded as 0. Women who scored between 1 and 27 were considered food insecure and were coded as 1.

Women’s ethnicity served as a predictor variable. The covariates were grouped into four sets:

- Demographic characteristics: age, education, marital status, birth(s) in the last five years, total household members, sex of the household head
- Economic characteristics: women’s property ownership status, employment/work status, household wealth index
- Cultural characteristics: women’s religion
- Geographic/Ecological characteristics: rural/urban, ecological zone, development region

An operational definition of all variables is provided in Table 1.

**Table 1:** List of variables used in the analysis

Household food insecurity (HFI)	A nine-item HFI scale was used to assess household food insecurity. In the past 12 months: (1) how frequently did you worry that your household would not have enough food? (2) how often were you or any household member not able to eat the kinds of foods you preferred because of a lack of resources? (3) how often did you or any household member have to eat a limited variety of foods due to a lack of resources? (4) how often did you or any household member have to eat some foods that you really did not want to eat because of a lack of resources to obtain other types of food? (5) how often did you or any household member have to eat a smaller meal than you felt you needed you felt you needed because there was not enough food? (6) how often did you or any household member eat fewer meals in a day because of lack of resources to get food? (7) how often was there with no food to eat of any kind in your household because of lack of resources to get food? (8) how often did you or any household member go to sleep at night hungry because there was not enough food? And (9) how often did you or any household member go a whole day and night without eating anything because there was not enough food? Answer categories for each item included never (0), rarely (1), sometimes (2), and often (3). A summated HFI scale ranged from 0 to 27. Respondents who answered “never” to all nine questions and maintained a zero summated scale score were coded as 0; those who scored 1 to 27 were coded as 1.
Ethnicity	Ethnicity had 11 categories. We recoded them into 7 meaningful dummy variables: Brahmin/Chhetri, Newar, Hill Indigenous, Terai Indigenous, Muslim, Dalit, and Other castes. In logistic regressions, Dalit served as the reference category.
Sex of the household head	If the head of the household was a female it was coded as 1; else = 0
Total household members	A continuous variable representing total household members living in the same household.
Age at interview	A continuous variable representing women’s age.
Women’s education	*Education* included four dummy variables: no education, primary education (pre-primary to the completion of 5th grade of schooling), secondary education (6th grade to the completion of 10th grade); and High School and above (beyond 10th grade). No education served as the reference category in regression.
Women’s marital status	Women who were married or living with partner were recoded as 1; all others including never in union, widowed, divorced or not living together were coded as 0.
Had birth(s) in the last 5 years	Women who had given at least one birth in the last five years were coded as 1 and those who had not given any birth during the same time were coded as 0.
Wealth index	NDHS has a five-level (1–5) wealth quantiles index (poorest, poorer, middle, richer & richest) variable by assigning each household the scores derived using principal component analysis based on the ownership of a wide range of goods (e.g., television, bicycle, car, housing characteristics such as source of drinking water, toilet facilities, and flooring materials). We used this variable as continuous.
Property ownership	If women owned a house or land they were coded as 1; else = 0
Employment/Work	If women worked aside from their house work, they were coded 1; else = 0.
Religion	Hindu women were coded as 1; else they were coded as 0.
Residence	Urban residence = 1; rural =0
Ecological zone	Based on the terrain, NDHS has divided Nepal into three regions: Mountain region of the north, middle hills, and Terai in the south. We dummy coded them into three variables. In logistic regression, Terai served as the reference group.
Development Region	*Development region* included 5 dummy coded administrative regions: Eastern (reference group), Central, Western, Midwestern and Far-western regions.

### Analysis strategy and diagnostic tests

First, the prevalence of food insecurity was examined using descriptive statistics. Next, the following multiple logistic regression (“logit”) model estimated the odds of experiencing food insecurity [53], where X_1_ is a vector of demographic characteristics, X_2_ is a vector of economic characteristics, X_3_ is a vector of cultural characteristics, and X_4_ is a vector of geo-ecological characteristics, and X_5_ is a vector of ethnicity for individual *i*:

$$ y=\left\{\begin{array}{c}0\  if\ food\ secure\\ {}1\  if\ food\ insecure\end{array}\right. $$

$$ Logit\ p\left({y}_i=1\right)={\beta}_0+{\beta}_1{X}_{i1}+{\beta}_2{X}_{i2}+{\beta}_3{X}_{i3}+{\beta}_4{X}_{i4}+{\beta}_5{X}_{i5} $$

In the equation above, *β*_0_ is the intercept and, *β*_1_, *β*_2_, *β*_3_, and *β*_4_ are the coefficients associated with each set of covariates and *β*_5_ is associated with the predictor variable (ethnicity). Nominal (e.g., ethnicity) and ordinal (e.g., education) and variables were discretized into a set of *k* indicator variables, with *k-1* included in estimating the equation to avoid perfect collinearity. The excluded category serves as the base category for interpretation of model results with respect to these variables. Analyses were conducted using probability weights and SAS version 9.2 procedures that account for complex survey design. Direct interpretation of nonlinear regression coefficients, as in a logit model, is challenging. Therefore, the model results have been expressed as odds ratios or the exponent of the log of the odds [54, 55].

The logit model was estimated using responses from 12,859 women with three cases excluded due to missing values in one of the predictor variables (religion). The variance inflation factors (VIF) were generated to diagnose multicollinearity among the independent variables. The highest-observed VIF was 2.7, and the lowest was 0.37, indicating a lack of serious multicollinearity [55]. Model fit was assessed using the Hosmer and Lemeshow goodness-of-fit test, a comparison of observed and predicted events, with unweighted data [54]. A large difference between observed and predicted frequencies results in a high χ^2^ value. An insignificant χ^2^ implies the model fits the data. Tests were also conducted to assess the predictive ability of the model. The results are reported under the goodness-of-fit test.

## Results

Descriptive statistics for all variables are shown in Table 2. Food insecurity is quite common—about 56% of all women and 76% of Dalit women of reproductive age had experienced HFI in the 12 months prior to the interview. One-third of the women had no education, and 77% were married. About 31% of the women had given birth in the last five years. Almost 57% were employed or worked for pay aside from housework. About 12% of the women identified themselves as Dalit, almost 21% as Hill indigenous, 10% as *Terai* indigenous, 5% Newar, 5% Muslim and 32% Brahmin/Chhetri. The latter is considered the privileged caste in Nepal.

**Table 2:** Weighted descriptive results using individual, nationally representative sample women of reproductive age (*n* = 12,862)^i^, Nepal, 2016.

Variables	Percentage	Mean	Std. Dev
% Food insecure	55.74		
Ethnicity			
% Dalit	12.41		
% Brahmin/Chhetri	31.66		
% Newar	04.97		
% Hill indigenous	20.95		
% Terai Indigenous	09.85		
% Muslim	05.00		
% Other	15.17		
Sex of the household head			
% Female	31.07		
Total household members (Mean)		05.46	2.80
Age of women at interview (Mean)		29.32	9.73
Women’s education			
% No education	33.28		
% Primary (1-5th grade)	16.72		
% Secondary (6-10th grade)	35.11		
% High School & above (>10th grade)	14.89		
% Married	76.77		
% Had birth(s) in the last 5 years	31.08		
Wealth Index (Mean)		03.12	1.39
% Owns property (home or land or both)	15.07		
% Employed/working	56.92		
Religion			
% Hindu	85.85		
Residence			
% Urban	62.76		
Ecological zone			
% Mountain	06.02		
% Hill	43.20		
% Terai	50.78		
Development Region			
% Eastern	22.55		
% Central	35.53		
% Western	20.19		
% Midwestern	12.83		
% Far-western	08.91		

^i^Unweighted sample size

Table 3 shows the results of the multivariable logistic regression model presented as adjusted odds ratios. Values greater than 1 indicate the factor is associated with increased risk of food insecurity while values below 1 indicate the factor is protective against food insecurity. Ethnicity was a significant predictor of food insecurity. In the model, Dalit was treated as the base category. All the odds ratios are below 1, so Dalits have a higher covariate-adjusted risk of food insecurity relative to all other ethnic groups. This relationship is statistically significant in all cases except for Newar. For example, Brahmin/Chhetri, the privileged caste group, were 46% less likely to be food insecure than Dalits even after accounting for other variables in the model (OR: 0.54; CI: 0.40–0.73). Similarly, Hill Indigenous, *Terai* Indigenous and Muslims were about 48, 47 and 45% less likely to be food insecure than Dalits, respectively. Alternately, the predicted odds of experiencing food insecurity for Dalit women in Nepal were 82, 85, 89 and 92% higher than Muslims, Brahmin/Chhetri, *Terai* Indigenous, and Hill Indigenous populations, respectively.

**Table 3:** Predicting women’s likelihood of experiencing food insecurity in Nepal, 2016

	All women of reproductive age (*n* = 12,859)^i^		
	Adjusted odds ratio	95% Confidence Limits	
Predictor variable		Lower	Upper
Ethnicity (Dalit = 0)			
Brahmin/Chhetri	0.54***	0.40	0.73
Newar	0.59	0.30	1.18
Hill Indigenous	0.52***	0.40	0.68
Terai Indigenous	0.53***	0.38	0.73
Muslim	0.55*	0.35	0.87
Other	0.47***	0.34	0.64
Covariates			
Sex of the household head (Female = 1; male = 0)	1.13	0.99	1.29
Total household members	0.98	0.95	1.01
Age of Women at Interview	0.99	0.99	1.00
Women’s education (no education = 0)			
Primary (1-5th grade)	0.75***	0.65	0.87
Secondary (6-10th grade)	0.61***	0.52	0.72
High School and above (>10th grade)	0.44***	0.36	0.53
Married (yes = 1; no = 0)	0.80**	0.68	0.94
Had birth(s) in the last 5 years (yes = 1; no = 0)	1.07	0.94	1.21
Wealth Index	0.55***	0.52	0.59
Owns property (1 = yes; 0 = no)	0.90	0.78	1.04
Employed/Working (1 = yes; no = 0)	0.97	0.86	1.09
Religion (Hindu = 1; else = 0)	1.01	0.80	1.29
Residence (Urban = 1; rural = 0)	0.98	0.79	1.20
Ecological zone (Terai = 0)			
Mountain	0.77	0.50	1.20
Hill	1.04	0.82	1.31
Development region (Eastern = 0)			
Central	1.60**	1.20	2.13
Western	0.90	0.68	1.20
Mid-western	1.91***	1.43	2.56
Far-western	1.46*	1.04	2.05
F value for Wald χ^2^	29.42***		
Max-rescaled R^2^	.26		
generalized R^2^	.20		
c	.77		
Hosmer and Lemeshow χ^2^ GFI	12.11(df = 8; *p* = .15)		

*p < .05; **p < .01; *** *p* < .001; ^i^Unweighted sample size

Among the demographic variables, education is associated with a reduced risk of food insecurity. Compared to women without any education, those with primary education (or up to the 5th grade) were 25% less likely to experience HFI, holding other variables constant (OR: 0.75; CI: 0.65–0.87). Those with secondary education (6th to 10th grade) were 39% less likely to experience food insecurity compared to their counterparts without any education (OR: 0.61; CI: 0.52–0.72) while women with a beyond 10th grade of education were 56% less likely to experience food insecurity compared to those without any education (OR: 0.44; CI: 0.36–0.53).

Being married was also a protective factor. Married women were 20% less likely to experience HFI than their unmarried counterparts even after accounting for all other factors (OR: 0.80; CI: 0.68–0.94). The number of household members and birth(s) in the last five years were not significantly associated with food insecurity. There was a significant inverse relationship between the household wealth index and food insecurity. A one unit increase in the household wealth index was associated with a 45% reduction in the predicted odds of food insecurity (OR: 0.55; CI: 0.52–0.59). Interestingly, women’s property ownership and paid work/employment aside from housework did not affect food insecurity after accounting for other variables. Also, the risk of food insecurity did not vary between Hindus and non-Hindus.

Geographically, residence in the Central, Mid-western and Far-Western regions were risk factors for food insecurity. Women residing in the Mid-western region of Nepal were most vulnerable, being 91% more likely to experience food insecurity than their counterparts in the Eastern region of Nepal (OR: 1.91; CI: 1.43–2.56) while holding other factors constant. Similarly, those from the Far-western development region of Nepal were 46% more likely to experience food insecurity than their counterparts from the Eastern Development region (OR: 1.46; CI: 1.04–2.05). Finally, women from the Central Development region were 60% more likely to be food insecure than women from the Eastern Development region (OR: 1.60; CI: 1.20–2.13).

### Goodness of-fit-test

The Hosmer and Lemeshow goodness-of-fit test was insignificant (χ^2^(8) = 12.11, *p*. = 0.15). The observed and model-predicted frequencies of food insecurity are not significantly different, implying the model fits the data well. We also plotted the Receiver Operating Characteristic (ROC) curve, a plot of the true positive prediction rate against the false positive prediction rate, to provide an overall assessment of predictive accuracy [54]. The area under this curve is referred to as the concordance index (c statistic). The c statistic can range from 0 to 1, where values below 0.5 are consistent with routine misprediction by the model, 0.5 suggests entirely random predictions of the response, and 1 indicates a perfect prediction of the response. The closer the value of c to 1, the higher the level of correct classification. The c statistic for the logistic regression model estimated here was 0.77, indicating a modest level of discrimination [54]. Additionally, to assess overall strength of the model, generalized R^2^ and Max-rescaled R^2^ were generated. They test the null hypothesis that all coefficients in the model are zero [53]. In the current study, the generalized R^2^ was .20 and the Max-rescaled R^2^ was .26, indicating that some of the regression coefficients are significantly different from zero and that the model has a modest predictive power.

## Discussion

The topic of food insecurity has been more widely studied in the developed nations [56]. This study documents the prevalence and correlates of food insecurity in a developing country, Nepal, using recent, nationally representative data. In this section, some of the key findings will be discussed.

First, ethnicity was an important determinant of food insecurity. Food insecurity was common among almost all ethnic groups. Strikingly, however, 76% of Dalit women were in food insecure households. The odds of a Dalit woman of childbearing age experiencing HFI were significantly higher than for women of nearly all other ethnic groups even after accounting for other relevant factors. These results align with other studies that have documented that HFI is substantially higher among Dalits in Nepal 38,39. They also suggest that social exclusion plays a role in food insecurity. Due to generations of caste-based discrimination, Dalits in Nepal have very low access to economic opportunities--education, employment, property ownership, and economic institutions. They are often concentrated in rural areas serving as landless agricultural laborers with high malnutrition among women and children [39]. Studies from India also suggest that food insecurity and malnutrition are particularly acute among Dalit women in that country [57, 58]. Some have suggested reviving the agriculture of indigenous food crops—sorghum, pulses, vegetables and animal sources of food—and increasing the consumption of these products among Indian Dalit mothers to improve their nutritional status [57, 58]. In Nepal, social policy has been directed toward reducing disparities between Dalits and other groups. Since 1997, the government has funded programs and activities aimed at improving the quality of life of Dalits. These initiatives include scholarship programs for secondary and higher education of Dalit children, income generation activities of Dalit men and women, and mass communication programs to raise public awareness on caste discrimination; these programs, however, are often poorly funded and implemented [39].

One option for social policy intervention may be to expand Nepal’s income transfer policies to specifically benefit the most food insecure populations. Low-income countries around the world, including Nepal, have been developing and expanding income transfer policies. For example, Nepal has been slowly building its Social Security Program since 1994/95 and has now instituted a universal old age (70+), disabled, and widows (60+) pension plan that transfers a set amount of monthly income to eligible elderly, disabled and widowed persons [59]. As Nepal prepares to tackle food insecurity among women of reproductive age, children, and minorities, perhaps benefit policies could specifically target Dalit women and their children. Such programs could be piloted in a district with a high concentration of food insecurity and proportionally large Dalit population. For example, according to the 2016 NDHS, the population of Baitadi District of the Far-Western Development Region was 41% Dalit, while nationally Dalits are about 12 to 14% of the population. Also, nearly 90% of the women from Baitadi had experienced food insecurity in the past 12 months.

Second, consistent with previous studies [13, 27], education is a protective factor for food security for women of reproductive age in Nepal. Only 35% of all women with education beyond 10th grade were food insecure. Among those with no formal education, 68% were food insecure. One possible path to increasing food security among women, then, is to increase the enrollment of girls in school and retain them at least until they complete high school or 12th grade of education. Early investment in the enrollment of girls, retention of these girls in school, and their advancement into the next grade level will likely improve women’s education and subsequently reduce HFI. In recent years, Nepal has made impressive efforts to increase girls’ enrollment in school. The “Girl Summit” of 2016 was committed to supporting the education of girls and boys by improving the school and community environments [60]. Nepal’s neighboring countries—India and Bangladesh—have launched financial incentive programs to increase demand for the enrollment and retention of girls in schools [61, 62]. Similar programs may increase the enrollment of female children in Nepal as well.

It is possible that educational attainment is a proxy for some other factor, such as household economic resources not captured in the current list of variables or strength of the social network. This analysis does not identify intermediate factors and their contributions to the reduction of food insecurity. Even if education is only indirectly related to food security, increasing educational achievement is still an important intervention strategy, as it should improve these intermediate outcomes. Future research in the context of countries such as Nepal might aim to clarify causal pathways.

Third, as expected, household wealth was a protective factor for food security. Policies could be designed to bolster the economic security of households with no or limited wealth. For example, in recent years, several developing countries in Africa have tested Unconditional Cash Transfer (UCT) programs. These initiatives make a targeted transfer without any behavioral requirements to reduce poverty and hunger immediately [63, 64]. Across sub-Saharan Africa alone, there are now over 123 UCT programs [65]. Studies assessing the impact of UCT find these programs improve dietary diversity and food security [66]. A study from Zambia comparing the impact of two government-run poverty alleviation programs using cluster randomized controlled trials found that UCT increased household per capita consumption expenditures by 20% and reduced food insecurity significantly [67]. In Burkina Faso, an evaluation of a UCT program, again using a cluster-randomized controlled trial, found a significant increase in the dietary intake of high-nutritional-value foods in young children between 14 and 27 months of age [68].

Nearly all evaluation studies have concluded that UCTs hold promise in reducing poverty and food insecurity. The primary arguments against such programs focus on their fiscal viability [69]. Nepal could test the concept of UCT in one of the high food insecure districts of the Far- or Mid-Western region (discussed in more detail subsequently) with particular attention to fiscal feasibility and cost-effectiveness. Also, there are many local non-governmental organizations and external developmental partners (EDPs)-- international governmental and non-governmental organizations-- working in Nepal to improve health, education, and agriculture sectors [70–72]. Perhaps, some of the EDPs could be specifically directed to work in food insecure districts and test new ideas such as the UCT.

Fourth, geography is a predictor of food insecurity in Nepal. In our study, food insecurity is most pronounced in the Mid-Western development region compared to the Eastern Development region, a finding consistent with previous research using 2011 NDHS data [40]. Out of Nepal’s 75 districts, six of the ten highest food insecure districts are in the Mid-Western Development Region. In these districts, food insecurity ranged from 83 to 100% of women of child-bearing age. These districts include: Kalikot (83%), Rolpa (89%), Dailekh (86%), Dolpa (94%), Jumla (91%) and Humla (100%). The remaining four districts are distributed in the Far-Western Development region (Baitadi, 90%), Central Development region (Rasuwa, 87% and Ramechhap, 85%) and Eastern Development region (Khotang (90%). As mentioned previously, these districts could serve as test cases for a UCT program.

Fifth, one surprising result in the current study is the lack of a statistically significant relationship between the gender of the household head and food insecurity in the multivariable model. One possibility is that women’s vital contribution as food producers may have buffered this relationship [73]. Our interest in women of childbearing age is partly motivated by previous findings that female-headed households are more susceptible to food insecurity [41]. In contrast, there was no statistically significant difference in food insecurity by the sex of household head. About 31% of women lived in a female-headed household. These household heads could be grandmothers, widows, divorced women, or married women whose husbands were not at home. One or more of these subtypes of households may be more susceptible to food insecurity. In turn, omitted factors could explain the differences between our study and previous research. Future studies using qualitative data might be able to better describe the food insecurity experiences of women who head households, further explaining the discrepancy.

Lastly, this study has strengths and limitations. A strength of this study is that it uses nationally representative data with very few missing cases, so the results are generalizable to the population. It also incorporates a more extensive food security measure than earlier studies of food security in Nepal. For the first time, the 2016 NDHS employed the full nine-item Household Food Insecurity Access Scale. The 2011 NDHS used only seven of the nine items. This has implications for monitoring progress toward attaining the Sustainable Development Goals (SDGs). In September 2015, the United Nations and its 193 member countries adopted *2030 Agenda for Sustainable Development*, which includes 17 SDGs to be attained by 2030 [74]. Goal 2 aims to eliminate hunger globally. Specifically, SDG 2.1 seeks to end hunger and ensure access to safe, nutritious, and sufficient food all year round for all people. SDG 2.2 intends to end all forms of malnutrition, stunting and wasting in children under five years of age, and ensure the nutritional needs of adolescent girls, pregnant and lactating mothers and older adults. This study focuses on a subpopulation—women of childbearing age—for whom food insecurity, because of the subsequent consequences for children, has broader implications. This analysis showed that ethnicity is associated with HFI even after accounting for some economic, social, and geographic factors among these women. As we move toward the 2030 Agenda, these findings provide baseline data to monitor progress toward the elimination of food insecurity among women of reproductive age in Nepal and offer potential vectors for intervention.

While using a strong data source, a limitation of this analysis is that it is cross-sectional and reflects correlational relationships only. Additional research, whether qualitative or using more advanced quantitative methods, is needed to make persuasive causal claims. The nine-item HFIAS employed in the 2016 NDHS to assess household food insecurity has received mixed evaluations. A review of nine studies from India has questioned the reliability of four items corresponding to anxiety (e.g., ‘worried’) about food and quality of food (e.g., ‘preferred food’, ‘limited variety’) [75]. Sethi and colleagues (2017) imply that the response to these items varies by culture, threatening validity. The current study used the entire scale to define food insecurity (see Table 1). If the criticisms of the scale are accurate, at least two issues arise. First, this study may have overestimated the actual level of food insecurity in the overall population of women of child-bearing age. If the differential interpretation of items is culturally patterned, the current estimates of disparities by ethnic group may be systematically biased. Second, the reference period of “past 12 months” for the nine items assessing food insecurity is a concern. Such a long timeframe increases the risk of recall bias, and also restricts us from examining the known seasonality of food insecurity in Nepal.

## Conclusions

This study is not the first to examine food insecurity in Nepal, though it is the first to consider women of child-bearing age specifically using the latest nationally representative sample. The results demonstrate that food insecurity among women of child-bearing age in Nepal is higher among Dalits even after accounting for other relevant characteristics. These findings are generally consistent with existing research, and the very high prevalence of food insecurity among Dalits suggests social exclusion plays an important role in experiences of food insecurity. The findings here should be useful to policymakers and social work practitioners as they decide on methods and target populations for interventions to achieve 2030 Sustainable Development Goals.

If universal policies are not possible, special attention is warranted to Dalits and those in Mid-western Nepal generally. The results also suggest that education and wealth-building are potential vectors for addressing food insecurity, though it is not possible to make clear causal claims from this cross-sectional study. As mentioned earlier, perhaps social policies could be piloted with a focus on building wealth among women with children in districts with a high concentration of food insecurity and proportionally large Dalit population such as Baitadi District of the Far-Western Development Region and Dolpa, Jumla or Humla districts of the Mid-Western Development region. Finally, as Nepal progresses toward attaining the SDG goals by 2030, public health and social work researchers can document progress against this baseline. In particular, do the disparities identified in this study linger even after efforts to reduce food insecurity or to improve one of the relevant protective factors, such as education?

## Data Availability

The 2016 NDHS data are available for public use with permission from the DHS program (for details see, https://dhsprogram.com/data/dataset/Nepal_Standard-DHS_2016.cfm?flag=0).

## Abbreviations

EDP: External Developmental Partner
HFI: Household food insecurity
HFIAS: Household Food Insecurity Access Scale
HIV: *Human immunodeficiency virus*
NDHS: Nepal Demographic and Health Survey
SDG: Sustainable Development Goals
UCT: Unconditional Cash Transfer
